# A longitudinal study over 40 years to study the metabolic syndrome as a risk factor for cardiovascular diseases

**DOI:** 10.1038/s41598-021-82398-8

**Published:** 2021-02-03

**Authors:** Lars Lind, Johan Sundström, Johan Ärnlöv, Ulf Risérus, Erik Lampa

**Affiliations:** 1 Department of Medical Sciences, Uppsala University Hospital, Uppsala University, 751 85 Uppsala, Sweden; 2 The George Institute for Global Health, University of New South Wales, Sydney, NSW Australia; 3 Division of Family Medicine and Primary Care, Department of Neurobiology, Care Sciences and Society, Karolinska Institutet, Huddinge, Sweden; 4 School of Health and Social Sciences, Dalarna University, Falun, Sweden; 5 Department of Public Health and Caring Sciences, Uppsala University, Uppsala, Sweden; 6 Uppsala Clinical Research Center, Uppsala University, Uppsala, Sweden

**Keywords:** Risk factors, Epidemiology, Genetics research, Myocardial infarction, Heart failure, Stroke, Metabolic syndrome

## Abstract

The impact of most, but not all, cardiovascular risk factors decline by age. We investigated how the metabolic syndrome (MetS) was related to cardiovascular disease (CVD) during 40 years follow-up in the Uppsala Longitudinal Study of Adult Men (ULSAM, 2,123 men all aged 50 at baseline with reinvestigations at age 60, 70, 77 and 82). The strength of MetS as a risk factor of incident combined end-point of three outcomes (CVD) declined with ageing, as well as for myocardial infarction, ischemic stroke and heart failure when analysed separately. For CVD, the risk ratio declined from 2.77 (95% CI 1.90–4.05) at age 50 to 1.30 (95% CI 1.05–1.60) at age 82. In conclusion, the strength of MetS as a risk factor of incident CVD declined with age. Since MetS was significantly related to incident CVD also at old age, our findings suggest that the occurrence of MetS in the elderly should not be regarded as innocent. However, since our data were derived in an observational study, any impact of MetS in the elderly needs to be verified in a randomized clinical intervention trial.

## Introduction

The metabolic syndrome (MetS) was first described in 1988 by Reaven and other research groups^1,2^. The prevalence of the syndrome ranges between 5–45% depending on the definition and age of the population, but despite this many studies have shown MetS to be associated with future cardiovascular (CV) morbidity and mortality in different populations around the world^3–17^.

The use of the MetS has been questioned^7^, mainly due to the fact that the risk of cardiovascular disease (CVD) associated with the syndrome is not greater than the sum of its individual components^15,16^. Nevertheless, MetS is a clinically useful descriptive term for overweight/obese subjects with several CV risk factors.

Using repeated measurements in the same population, it has previously been described that some CV risk factors lose in strength as risk factors during the life-span, while other risk factors retain their strength also at a higher age^18,19^. We have recently expanded this analysis to cover repeated examinations over 40 years, and found that most risk factors lose in strength as risk factors by ageing, but low-density lipoprotein (LDL) cholesterol was a powerful risk factor for myocardial infarction also at age 82, and fasting glucose and body mass index (BMI) were important risk factors for heart failure at high age^20^.

In the present study, we evaluated the impact of ageing on the strength of MetS as a risk factor for CVD using the Uppsala Longitudinal Study of Adult Men (ULSAM) with repeated examinations during a more than 40-year follow-up period, with the hypothesis that the power of MetS to predict CVD would decline with ageing, but still be significant also at high age. As a secondary aim, we also investigated the strength of MetS on the risk of the specific CV diseases included in the definition of CVD; myocardial infarction, ischemic stroke and heart failure.

## Results

In the present study, subjects with a history of myocardial infarction, stroke or heart failure at baseline at age 50 were excluded from further analysis (n = 30). When also individuals with missing data on risk factors included in the definition of the syndrome were excluded, the baseline sample at age 50 consisted of 2,123 men. The vast majority of missing data were due to a failure to measure lipids during a period of time. No significant difference in BMI or systolic blood pressure was seen between subjects with and without missing lipid data.

At age 50, the prevalence of MetS was 13%, but increased to 35% at age 60. The prevalence thereafter declined to 23% at age 70 and remained essentially at that level also at old age. An increase in BMI by 1.2 kg/m^2^ from age 50 to 70 was noted, but was fairly stable after age 70. A gradual increase in systolic blood pressure was noted by 17 mmHg up to age 77, while an increase in fasting glucose was seen up to 82 years. The lipids declined over the follow-up period (see Table 1 for details).

**Table 1:** Number of subjects at risk, number of individuals withdrawn from analysis due to prevalent cardiovascular disease (CVD) at the examination, prevalence of the metabolic syndrome (MetS), prevalent cardiovascular disease, cardiovascular risk factors and medications at the time of the different investigations.

		50 years	60 years	70 years	77 years	82 years
n at risk		2123	1763	1073	669	395
Excluded due to prevalent CVD		30	73	141	174	130
Prevalence of MetS (%)		13	35	23	21	24
Fasting glucose (mmol/l)		5.5 (0.9)	5.5 (1.4)	5.7 (1.4)	5.8 (1.3)	5.9 (1.2)
Triglycerides (mmol/l)		1.93 (1.24)	1.66 (0.70)	1.45 (0.77)	1.38 (0.69)	1.39 (0.68)
HDL-cholesterol (mmol/l)		1.36 (0.38)	1.28 (0.24)	1.28 (0.35)	1.31 (0.32)	1.20 (0.29)
LDL-cholesterol (mmol/l)		5.26 (1.19)	4.43 (0.66)	3.89 (0.90)	3.47 (0.85)	3.39 (0.84)
BMI (kg/m^2^)		25.0 (3.1)	25.4 (3.2)	26.2 (3.4)	26.3 (3.4)	26.0 (3.4)
Systolic blood pressure (mmHg)		133.0 (18.0)	142.5 (19.6)	146.7 (18.5)	150.5 (19.9)	145.0 (17.4)
Antihypertensive medication (%)		4	19	35	42	54
Lipid lowering medication (%)		0	6	9	17	21
Antidiabetic medication (%)		1	2	6	9	10
**Exercise habits (frequency in % of number at risk)**						
1		15	11	4	8	15
2		37	53	35	35	36
3		43	32	55	52	44
4		5	4	6	4	5
Education level (frequency in % of number at risk)		< 10 years: 63 10–12 years: 26 > 12 years: 11				

Definitions of the exercise habits groups:

1. Do you spend most of your time reading, watching TV, going to the cinema, or engaging in other, mostly sedentary activities?

2. Do you often go walking or cycling for pleasure?

3. Do you engage in any active recreational sports or heavy gardening at least 3 h every week?

4. Do you regularly engage in hard physical training or competitive sport?

HDL, high-density lipoprotein; LDL, low-density lipoprotein; BMI, body mass index.

### Age related association between MetS and CVD

**Figure 1:**
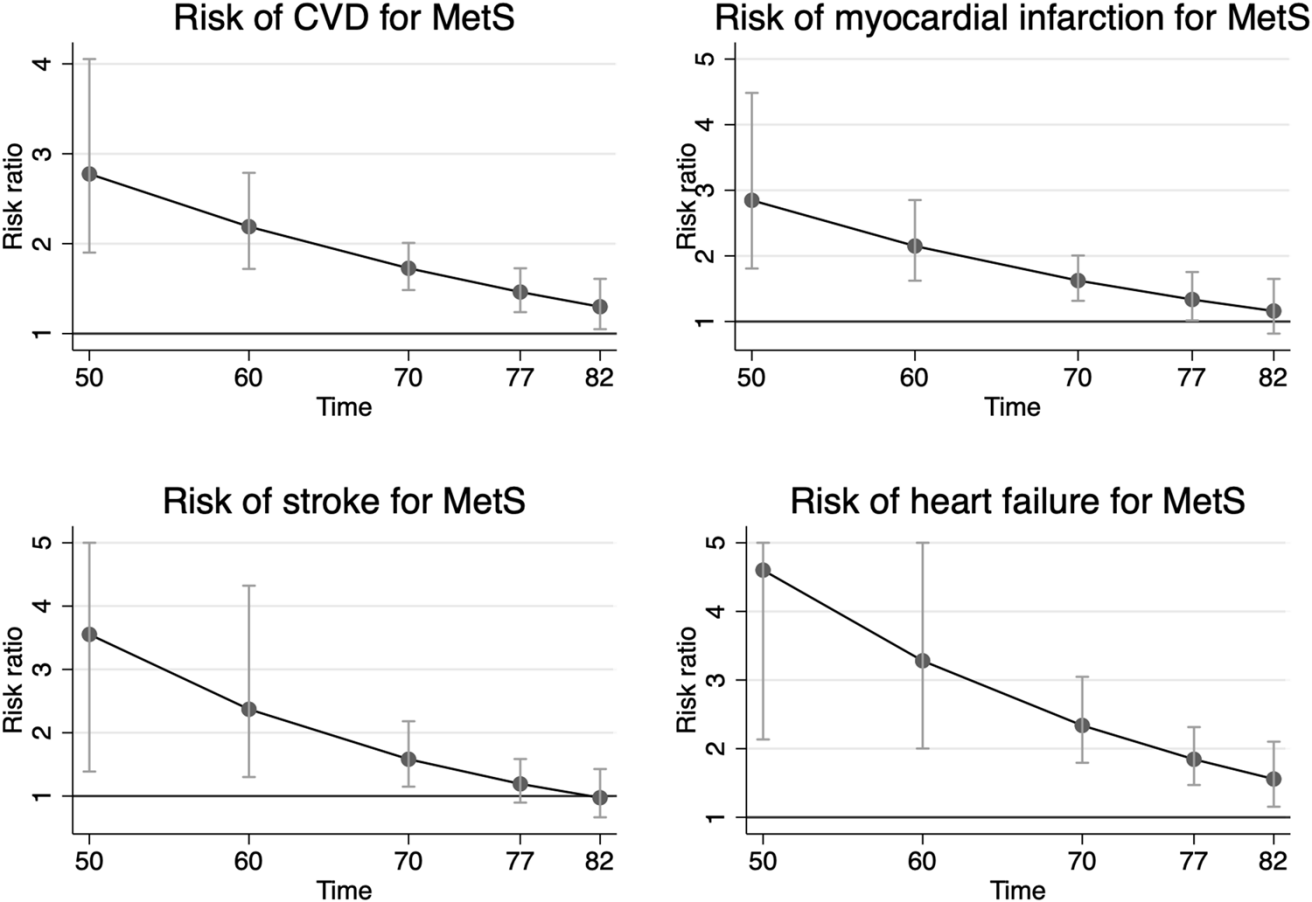
Risk ratio and 95% confidence interval for the metabolic syndrome (MetS) regarding incident cardiovascular disease (CVD, combined end-point of the other three outcomes), myocardial infarction, ischemic stroke and heart failure. The RRs were adjusted for LDL cholesterol, education level (years in school), leisure time exercise habits, smoking and use of medication against dyslipidaemia. The graphs were created using STATA16^33^.

As could be seen in Fig. 1, the strength of the association between MetS and incident CVD declined with ageing for the combined end-point CVD, as well as for its three components, myocardial infarction, ischemic stroke and heart failure. For CVD, the risk ratio declined from 2.77 (95%CI 1.90–4.05) at age 50 to 1.30 (95%CI 1.05–1.60) at age 82, being still significant at age 82 (*p* = 0.016).

For heart failure, MetS was a significant risk factor up to age 82. For myocardial infarction, MetS was a significant risk factor up to age 77. For stroke, MetS was only significant up to 70 years of age.

Detailed information is given in Table 2.

**Table 2:** Risk ratio (RR), 95%CI and *p*-value for the risk of incident cardiovascular disease (CVD, combined end-point), myocardial infarction, ischemic stroke and heart failure for prevalent metabolic syndrome (MetS) at different ages. The RRs were adjusted for LDL cholesterol, education level (years in school), leisure time exercise habits, smoking and use of medication against dyslipidaemia.

Age	RR	95%CI lower	95%CI upper	*p*-value
**CVD**				
50	2.77	1.90	4.05	1.24e-07
60	2.18	1.71	2.78	2.05e-10
70	1.72	1.48	2.00	1.56e-12
77	1.46	1.24	1.72	7.35e-06
82	1.30	1.05	1.60	0.016
**Myocardial infarction**				
50	2.87	1.81	4.48	6.31–06
60	2.15	1.62	2.85	1.02e-07
70	1.62	1.31	2.03	6.60e-06
77	1.33	1.02	1.75	0.038
82	1.16	0.81	1.65	0.40
**Ischemic stroke**				
50	3.55	1.38	9.10	0.0082
60	2.37	1.30	4.32	0.0048
70	1.58	1.14	2.18	0.0050
77	1.19	0.89	1.58	0.22
82	0.97	0.66	1.42	0.89
**Heart failure**				
50	4.60	2.13	9.91	9.85e-05
60	3.28	2.00	5.37	2.40e-06
70	2.33	1.79	3.04	3.17e-10
77	1.84	1.47	2.31	1.16e-07
82	1.55	1.15	2.10	3.73e-03

**Figure 2:**
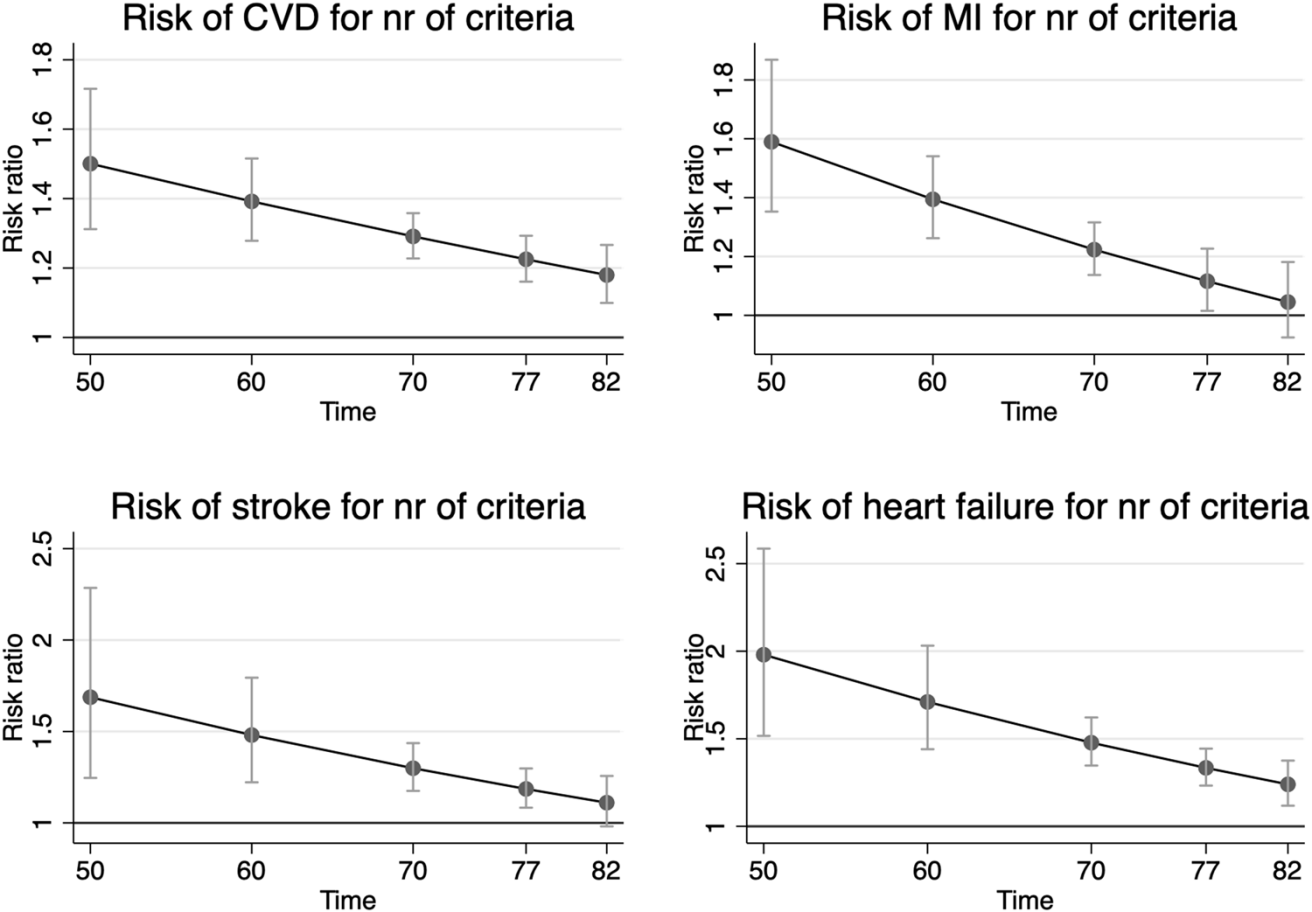
Risk ratio and 95% confidence interval for the number of metabolic syndrome components regarding incident cardiovascular disease (CVD, combined end-point of the other three outcomes), myocardial infarction, ischemic stroke and heart failure. The RRs were adjusted for LDL cholesterol, education level (years in school), leisure time exercise habits, smoking and use of medication against dyslipidaemia. The graphs were created using STATA16^33^.

As could be seen in Fig. 2, similar as was seen for MetS as a binary variable, the risk of CVD for a number of MetS components declined with ageing for the combined end-point CVD, as well as for its three components, myocardial infarction, ischemic stroke and heart failure. For CVD, the risk ratio declined from 1.50 for each increase in 1 component (95%CI 1.31–1.71) at age 50 to 1.17 (95%CI 1.09–1.26) at age 82, being still significant at age 82 (*p* = 4.5*10^–6^). Number of components was significant up to age for heart failure, but only up to age 77 for myocardial infarction and stroke.

Detailed information is given in Table 3.

**Table 3:** Risk ratio (RR), 95%CI and p-value for the risk of incident cardiovascular disease (CVD, combined end-point), myocardial infarction, ischemic stroke and heart failure for the number of metabolic syndrome (MetS) components at different ages.

Age	RR	95%CI lower	95%CI upper	*p*-value
**CVD**				
50	1.50	1.31	1.72	3.14e-09
60	1.39	1.28	1.52	2.51e-14
70	1.29	1.23	1.36	3.49e-23
77	1.23	1.16	1.29	1.66e-13
82	1.18	1.10	1.27	4.55e-06
**Myocardial infarction**				
50	1.59	1.35	1.87	1.89e-08
60	1.39	1.26	1.54	6.45e-11
70	1.22	1.14	1.32	6.06e-08
77	1.12	1.02	1.23	0.022
82	1.05	0.92	1.18	0.47
**Ischemic stroke**				
50	1.69	1.25	2.29	0.00072
60	1.48	1.22	1.79	0.000062
70	1.30	1.18	1.44	3.23e-07
77	1.19	1.08	1.30	0.00022
82	1.11	0.98	1.26	0.096
**Heart failure**				
50	1.98	1.52	2.59	5.22e-07
60	1.71	1.44	2.03	9.53e-10
70	1.48	1.35	1.62	1.73e-16
77	1.33	1.23	1.44	9.24e-13
82	1.24	1.12	1.38	.0000485

RR is given for a one risk factor increase. The RRs were adjusted for LDL cholesterol, education level (years in school), leisure time exercise habits, smoking and use of medication against dyslipidaemia.

## Discussion

The present study showed that the strength of the association between MetS and future CVD declined with ageing, but was still significant also at old age. This was seen both when MetS was used as a binary variable, as well as when we used the number of MetS components.

A number of previous studies have investigated the strength of the association of MetS as a “unified” risk factor for CVD and generally found the relative risk to be approximately doubled in individuals with MetS^3–17^. This is well in line with the present study, in which we found the risk ratio for CVD to decline from slightly above 2 at age 60 to slightly below 2 at the age of 70. The mean age of the populations previously investigated has usually been in that age- range.

Not surprisingly, the strength of the association between MetS and incident CVD declined with ageing given that we recently reported that the strength of the majority of the risk factors included in the MetS on subsequent CVD also declined with age^20^. However, this is the first study that has investigated the association between MetS and CVD in a systematic fashion over several decades. Furthermore, given the long follow-up and thereby a fairly large number of incident cases of the major CVD, we could also investigate the strength of the association between MetS incident myocardial infarction, ischemic stroke and heart failure as separate diseases.

In this analysis, the strength of the association between MetS and risk of the three CVDs at high age was most evident for heart failure and least evident for ischemic stroke, with myocardial infarction in between. Since blood pressure is the major risk factor for ischemic stroke it seems likely that the “unified” MetS, also including four other risk factors, is not as powerful a risk factor as when used to predict the other two outcomes. Since LDL is a major risk factor for myocardial infarction and is not included in the MetS, that fact also limits the power for MetS as a “united” risk factor for myocardial infarction.

In a previous report from the ULSAM study^20^, we have presented a detailed analysis of how ageing affects the strength of different risk factors included in MetS on the major CVDs. As could be seen in Table 3 and Fig. 4 in that paper, all of the evaluated risk factors were associated with myocardial infarction at middle-age (age 50 and 60), except for triglycerides. At middle-age, only blood pressure, glucose and smoking were risk factors for ischemic stroke, while obesity, HDL-cholesterol, blood pressure and smoking were the major risk factors for heart failure. Thus, although the MetS as such was related to all three major CVDs at middle age in the present study, the strength of the individual risk factors included in MetS varied between the three diseases. The most evident example of this is that blood pressure is by far the most important risk factor for ischemic stroke.

At old age (age 77 and 82), the strength of most individual risk factors has declined, but in the elderly LDL-cholesterol, not included in MetS, is the major risk factor for myocardial infarction, while the MetS variables blood pressure, glucose and HDL-cholesterol were of borderline significance. Regarding ischemic stroke, the strength of blood pressure declined markedly during ageing, while the strength of HDL- and LDL-cholesterol were powerful at old age. The strength of blood pressure as a risk factor declined markedly during ageing also in the case of heart failure, while obesity and glucose were the major risk factors at old age. Thus, compared to middle-age, the relative strength of the components included in MetS changed so that different parts of MetS are major risk factors at different ages.

The strength of the present study is the long follow-up period with repeated measurements of MetS enabling the present evaluation with a good power also for individual outcomes.

The major limitation is that the observational part is only performed in Swedish males and therefore has to be repeated in women, other ethnic groups and geographical locations to increase generalizability. In a middle-aged and elderly population, the prevalence of MetS was higher in males compared to females and the associations between fat mass and fat distribution (measured as waist-hip ratio) and occurrence of MetS were steeper in men than women^21^. The prevalence of MetS also differs between ethnic groups and geographical locations. For example, in India the prevalence of MetS was estimated to be 50% in middle-aged subjects according to a recent meta-analysis^22^, while the corresponding prevalence in China was 26% in another meta-analysis^23^ and around 20% in Japan^24^. In the US National Health and Nutrition Examination Survey, differences in the prevalence of MetS was described amongst ethnic groups^25^. Thus, given the differences in prevalences of MetS in different ethnic groups and geographical locations it is not given that results obtained in Swedish men applies to the rest of the world.

In this longitudinal study over 40 years from middle-age to old age, many subjects died or could not attend due to co-morbidities that occurred during the long follow-up period. This is an unavoidable fact that always complicate studies with a long follow-up period, especially if the follow-up includes old age. Since we therefore will have a selection bias of the fittest, a part of the decline in strength of MetS as a risk factor will for sure be due to this phenomenon. However, some risk factors persist or even increase in impact during ageing, like LDL-cholesterol for myocardial infarction and obesity and elevated glucose for heart failure^20^. Thus, even if the survival bias as such produce a decline in the strength of MetS and most risk factors included in MetS, there are exceptions from this major effect.

In conclusion, the strength of MetS as a risk factor of incident CVD declined with age. Since MetS was significantly related to incident CVD also at old age, out finding suggest that the occurrence of MetS in the elderly should not be regarded as innocent. However, since our data were derived in an observational study, any impact of MetS in the elderly needs to be verified in a randomized clinical intervention trial.

## Methods

### Study sample

In 1970 to 1973, all men born in 1920 to 1924 and residing in the county of Uppsala were invited to a health survey (at age 50) aimed at identifying risk factors for cardiovascular disease; 82% of the invited men participated (n = 2,322). The design and selection criteria for the cohort have been described previously^26,27^. Reinvestigations with physical examinations of the cohort were performed at ages 60 (n = 1836), 70 (n = 1214), 77 (n = 834), and 82 years (n = 525).

Written informed consent was obtained from all participants and the Uppsala University Ethics Committee approved the study, which was carried out in accordance with relevant guidelines and regulations.

### Baseline examinations

The examination at age 50 has been described in detail previously^26,27^. Blood samples for fasting concentrations were drawn in the morning after an overnight fast. Cholesterol and triglyceride concentrations in serum, and high-density lipoprotein (HDL) were assayed by enzymatic techniques. LDL cholesterol was calculated by Friedewald’s formula. Fasting blood glucose was determined by an oxidase method. Supine systolic and diastolic blood pressures were measured twice in the right arm after 10 min rest, and means were calculated. The physical examinations at the reinvestigations were performed essentially in the same way as at age 50. Leisure time physical activity was defined by the following four questions, being the same at all investigations:

1. Do you spend most of your time reading, watching TV, going to the cinema, or engaging in other, mostly sedentary activities?
2. Do you often go walking or cycling for pleasure?
3. Do you engage in any active recreational sports or heavy gardening at least 3 h every week?
4. Do you regularly engage in hard physical training or competitive sport?

At age 50, the participants were asked how many years they have spent in school/university.

### Metabolic syndrome

MetS was defined according to the harmonized criteria^28^ with some modifications. Three of the following five criteria should be fulfilled to be defined as an individual with MetS, while fulfilling two or less of these criteria was defined as an individual without MetS. (1) Blood pressure ≥ 130/85 mmHg or antihypertensive treatment, (2) fasting plasma glucose ≥ 5.5 mmol/l or use of glucose lowering drugs, (3) plasma triglycerides ≥ 1.7 mmol/l, (4) HDL cholesterol < 1.0 mmol/l in men and < 1.3 in women. (5). Since waist circumference was not measured at ages 50 and 60 in most subjects, we used data from 480 men at age 50 to calculate the BMI corresponding to a waist of 102 cm (29.4 kg/m^2^) and used this value at all ages in order to be comparable^14^. At age 70, at which both BMI and waist circumference were measured, only one subject was misclassified if BMI 29.4 kg/m^2^ was used instead of a waist circumference of 102 cm.

### Endpoint definitions

Date and cause of death were obtained from the Swedish Cause of Death Register. Date and cause of hospitalization were obtained from the Swedish Hospital Discharge Register in all individuals, not only those attending the re-examinations. The register data on diagnoses were updated annually. We evaluated three major CV diseases; acute myocardial infarction (ICD-8 code 410, ICD-9 code 310, or ICD-10 code I20), ischemic stroke (ICD-8 codes 431, 433-436, ICD-9 code 431, 433-436, ICD-10 code I63-I66) or heart failure using data from the Swedish Hospital Discharge Register. Combining data from the Swedish Cause of Death Registry and the Swedish Hospital discharge register is an efficient, validated alternative to revised hospital discharge notes and death certificates for both coronary heart disease and stroke^29^. Previous studies suggest that the accuracy of the heart failure diagnosis in the Swedish hospital discharge register has lower validity^30^ when including all diagnosis positions. Therefore, we performed extensive medical chart review in order to promote the highest quality of the diagnosis of heart failure and to include as many correctly classified heart failure events as possible. In short, as a possible diagnosis of heart failure, we considered ICD heart failure codes 427.00, 427.10, 428.99 (ICD-8), 428 (ICD-9), I50 (ICD-10) and hypertensive heart disease with heart failure, I11.0 (ICD-10) from the Swedish Hospital Discharge Register. The medical records from all relevant hospitalizations for heart failure were reviewed by one experienced physician (L.L.), who, blinded to the baseline data, classified the cases as definite, questionable, or miscoded. Only the definite cases were used in the following analyses. The classification relied on the definition proposed by the European Society of Cardiology^31^.

### Statistical analyses

To model the associations with MetS and the different outcomes, we used Poisson models with interactions between MetS and age to account for possible time-varying associations. Although the Cox model, which is the most common method used to analyse time-to-event data, can be extended to accommodate time-varying effects, the Poisson model allows for direct parametric modelling and estimation of the baseline rate which in this study was ageing^32^.

Follow-up time for each individual was further split into 1-year intervals in which we assumed that the rate was constant. Age was modelled with restricted cubic splines with knots placed at the 5th, 35th, 65th, and 95th percentiles of the age distribution, conditioned on the occurrence of an event. Two models were fitted for each outcome with MetS included both as a binary variable with categories MetS and No MetS and an ordered variable counting the number of individual components (0–5). Adjustment was made for LDL cholesterol, education level (years in school), leisure time exercise habits, smoking and use of medication against dyslipidaemia. LDL cholesterol was modelled using restricted cubic splines with three knots placed at the 10th, 50th, and 90th percentile of the LDL distribution. Non-linear terms for LDL were either kept or deleted based on a test of all non-linear terms, where the null hypothesis tested was that all regression coefficients corresponding to the non-linear terms were equal to zero. Non-linear terms for age were kept in all models and interactions were restricted to include linear terms only. Also included in the models were offsets equal to the natural logarithm of follow-up time for each individual.

We used multiple imputation to create and analyse 20 multiply imputed datasets. Incomplete variables were imputed under fully conditional specification. Model parameters were estimated with the Poisson model described above to each complete dataset separately. The estimates and their standard errors were then pooled using Rubin’s rules.

Data on LDL and HDL cholesterol, as well as triglycerides, were missing in a number of individuals at the examination at 60 years (68%, 88% and 88% respectively). Those values were imputed using multiple imputation to create and analyse 20 multiply imputed datasets. Incomplete variables were imputed under fully conditional specification. Model parameters were estimated with the Poisson described above to each complete dataset separately. The estimates and their standard errors were then pooled using Rubin’s rules. This described statistical procedure is the same as we previously have used and described in a previous paper^20^, although the exposure and confounding variables are different.

The number at risk for the combined end-point CVD is the total number of participants at the survey minus the subjects with essential missing data for calculation of MetS, minus those with prevalent CVD at that particular survey.

The description of the statistical methods used in the present study has previously been given in reference 20.

These analyses were made using R version 3.2.4 (R: A language for statistical computing, R core team, R foundation for statistical computing, Vienna, Austria, https://www.R-project.org) and the rms and Epi packages. Figures 1 and 2 were created in STATA16^33^.

## Data Availability

The data that support the findings of this study are available on request from the corresponding author.
